# Association Between Depression and Risk of Incident Cardiovascular Diseases and Its Sex and Age Modifications: A Prospective Cohort Study in Southwest China

**DOI:** 10.3389/fpubh.2022.765183

**Published:** 2022-03-30

**Authors:** Lisha Yu, Yun Chen, Na Wang, Kelin Xu, Chenghan Wu, Tao Liu, Chaowei Fu

**Affiliations:** ^1^Guizhou Center for Disease Control and Prevention, Guiyang, China; ^2^School of Public Health, Key Laboratory of Public Health Safety, National Health Commission Key Laboratory of Health Technology Assessment, Fudan University, Shanghai, China

**Keywords:** the Patient Health Questionnaire-9 (PHQ-9), depression, cardiovascular disease, effect modification, cohort study

## Abstract

To examine possible associations between depression and cardiovascular disease (CVD) incidence and whether demographic factors modified those associations in the Chinese population. This prospective cohort study comprised 7,735 adults aged 18 years or older in Guizhou, China from 2010 to 2020. The Patient Health Questionnaire-9 (PHQ-9) was used to measure the prevalence of depression. Cox proportional hazard models were used to estimated hazard ratios (HRs) and 95% confidence intervals (95%CIs) of depression and incident CVD. We identified 215 CVD cases (including 28 acute myocardial infarction (AMI) and 197 stroke cases) during an average follow-up of 7.07 years. In the multivariable-adjusted model, baseline PHQ-9 score was associated with incident CVD, AMI, and stroke. The HR per 1-SD increase for PHQ-9 score was 1.14 (95%CI: 1.03, 1.26) for CVD, 1.26 (95%CI: 1.01, 1.57) for AMI, and 1.12 (95%CI: 1.01, 1.25) for stroke. Compared with participants without depression, those with any mild or more advanced depression had a higher risk of incident CVD (HR: 1.69, 95%CI: 1.08, 2.64) and AMI (HR: 3.36, 95%CI: 1.17, 10.56). Associations between depression with CVD and stroke were suggested to be even stronger among women and participants aged <65 years (*P* for interaction <0.05). The effect of depression on stroke tended to be preserved in non-smokers. Depression was associated with a higher risk of incident CVD, AMI, and stroke in adults of Southwest, China, particularly in women, participants aged <65 years, and non-smokers. These findings highlighted the importance and urgency of depression healthcare.

## Introduction

Depression is a leading cause of disability, with more than an estimated 264 million people affected worldwide (1). Previous studies have reported that depression is consistently associated with a higher risk of adverse cardiovascular disease (CVD). In a meta-analysis of 28 prospective cohort studies, Pan et al. (2) reported a pooled adjusted hazard ratio (HR) of 1.45 for incident stroke associated with baseline depression. Another systematic review reported that people with major depression have a 56% higher risk of developing ischemic heart disease (IHD), with depression accounting for 2.95% of the disability-adjusted life years associated with IHD (3). However, the causal relationship between depression and CVD is still questionable, while the previous positive association was largely based on cross-sectional studies, cohort studies with short follow-up durations, or with inadequate adjustment of potential confounding factors. Also, the results had been inconsistent between different sociodemographic strata, such as men and women. For example, a meta-analysis found similar associations between depression and stroke in both men and women (4), while a study in Sweden suggested that the effect of depression on stroke was higher in men compared with women (5). The previous study has found a stronger association between depression and stroke in participants aged <65 years but not in participants ≥65 years (6). Therefore, more prospective cohort studies are still needed to examine whether the association between depression and CVD differs over sociodemographic factors.

To our knowledge, very few prospective cohort studies have been conducted on this issue among Chinese (7–9), in which three cohort studies covered middle-aged and older Chinese adults (7–9), and studies including younger adults in China were still not reported so far. In this study, we used data from a prospective cohort study in Southwest China to investigate whether depression was associated with CVD in adults and test whether those associations were modified by sociodemographic factors.

## Materials and Methods

### Study Design and Population

The Guizhou Population Health Cohort Study (GPHCS) was a prospective community-based cohort in Guizhou province located in Southwest China. A total of 9,280 residents was enrolled from 48 townships of 12 districts (or counties) in this cohort from 2010 to 2012 using a multistage proportional stratified cluster sampling method. The inclusion criteria included residents aged 18 years or older, who had no plan to move out and completed survey questionnaire and blood sampling. All participants were followed up for major chronic diseases and vital status through a repeated investigation from 2016 to 2020 with the loss to follow-up rate of 12.04%. A total of 428 participants were further excluded for this analysis, including 44 with a history of CVD, 214 without reliable information on CVD status at follow-up, and 170 without sufficient information on depression at baseline. This study was approved by the Institutional Review Board of Guizhou Center for Disease Control and Prevention (No. S2017-02). All participants signed the written informed consent.

### Assessment of Depressive Symptoms

The Patient Health Questionnaire-9 (PHQ-9) with a 9-question depression scale, was used to screen for the presence and severity of depressive symptoms according to the Diagnostic and Statistical Manual of Mental Disorders-IV criteria (DSM-IV) (10). Subjects were asked to respond to each symptom by rating the best statement applied over the past 2 weeks, using a score from zero to three (ranging from “not at all” = zero, “several days” = one, “more than half the days” = two, or “nearly every days” = three). Given a range of total scores between 0 and 27, the higher score indicated the greater severity of depressive symptoms. They were divided into three categories according to the PHQ-9 scores (0, no depression; 1 to 4, minimal depressive symptoms; and ≥5, mild or more advanced symptoms as depression) (11). The Chinese version of the PHQ-9 has demonstrated high reliability and validity (12).

### Ascertainment of Incident CVD Events

The study outcome was self-reported incident CVD events. Incident CVD events were assessed by the following standardized questions: “Have you been diagnosed with cerebral hemorrhage by a doctor?”, “Have you been diagnosed with subarachnoid hemorrhage by a doctor?,” “Have you been diagnosed with cerebral infarction by a doctor?”, or “Have you been diagnosed with acute myocardial infarction (AMI) by a doctor?” Participants who reported cerebral hemorrhage, subarachnoid hemorrhage, or cerebral infarction during the follow-up period were defined as having an incident stroke, and those who reported the above symptoms or AMI were defined as having incident CVD. All deaths were confirmed by the Death Registration Information System and Basic Public Health Service System, and deaths from AMI or stroke were considered as incident CVD cases.

### Covariates

Information on the covariates was collected by trained health workers using a structured questionnaire via a face-to-face interview, including sociodemographic characteristics (age, sex, ethnicity, education, marriage status, and occupation), lifestyle (smoking status, alcohol use, and physical activity), history of chronic diseases (type 2 diabetes (T2DM), hypertension, and dyslipidemia), and use of medications for T2DM, hypertension, and dyslipidemia. Height, body weight, and blood pressure were measured by trained health workers. Current smoker was defined as smoking at least one cigarette or other tobacco product a day for 12 months or more. Alcohol use was defined as drinking at least one time a week for 12 months or more. Physical activity was defined as having moderate or vigorous physical activity at least 10 min every time for one or more times per week. Body mass index (BMI) was calculated as body weight in kilograms divided by square height in meters (kg/m^2^). Venous blood samples were obtained from participants after at least 8 h overnight fast to measure fasting plasma glucose (FPG), total cholesterol (TC), triglycerides (TG), high-density lipoprotein cholesterol (HDL), and low-density lipoprotein cholesterol (LDL-C). A 2-h oral glucose tolerance test (OGTT) with 75 g of glucose was carried out for participants. T2DM was defined if participants met either of the following criteria: (1) self-reported doctor diagnosis of diabetes or use of anti-diabetic medications; (2) FPG ≥7.0mmol/L; (3) OGTT ≥11.1 mmol/L; (4) HbA1c ≥6.5% (13). Hypertension was defined as who met either of the following criteria: (1) systolic blood pressure ≥140 mmHg and/or diastolic blood pressure ≥90 mmHg; and/or (2) self-reported doctor diagnosis of hypertension or use of hypertension medications (14). Dyslipidemia was defined as who met either of the following criteria: (1) self-reported doctor diagnosis of dyslipidemia or use of lipid regulating drugs; (2) high TC: TC ≥6.22 mmol/L; (3) high TG: TG ≥2.26 mmol/L; (4) low HDL-C: HDL-C <1.04 mmol/L; 5) high LDL-C: LDL-C ≥4.14 mmol/L (15).

### Statistical Analysis

Data were described as means and SDs for continuous variables, and as frequencies and percentages for categorical variables. Baseline characteristics are summarized according to depression status and compared using the analysis of variance or the Chi-square test. The person-years (PYs) of follow-up were calculated for each participant from the date of enrolling the cohort to the date of the CVD diagnosis, death, or the date of follow-up, whichever came first. The associations of depression with CVD, AMI, and stroke were estimated using Cox proportional hazards regression models. Two models were estimated: (1) Model 1: age (<30, 30–39, 40–49, 50–59, 60–69, ≥70) and sex were adjusted; (2) Model 2: the variables in model 1 plus ethnicity (Han Chinese or non-Han Chinese), education (<9 or ≥9 years), marriage status (married or other), occupation (framer or other), smoking status (current smoker or non-smoker), alcohol use (yes or no), physical activity (yes or no), BMI, history of T2DM (yes or no), history of hypertension (yes or no), and history of dyslipidemia (yes or no) were adjusted. To assess the robustness of the results, the following sensitivity analyses were performed: (1) We repeated Model 2 after excluding participants who were followed up less than 2 years, and (2) Considering the competing risk of death, a competing risk model was also fitted. The potential effect modifications by age (<65 or ≥65 years old), sex, smoking status, alcohol use were estimated by (1) including multiplicative interaction terms in the multivariable Cox models; (2) fitting stratified models. We used the Schoenfeld residuals to test the assumption of hazard proportionality in Cox regression models and found no evidence of non-proportionality (*P* ≥ 0.05). Two-sides *P* < 0.05 was considered statistically significant. All analyses were performed in R software (Version 4.0.3; R Foundation for Statistical Computing, Vienna, Austria).

## Results

### Study Participants

**Figure 1:**
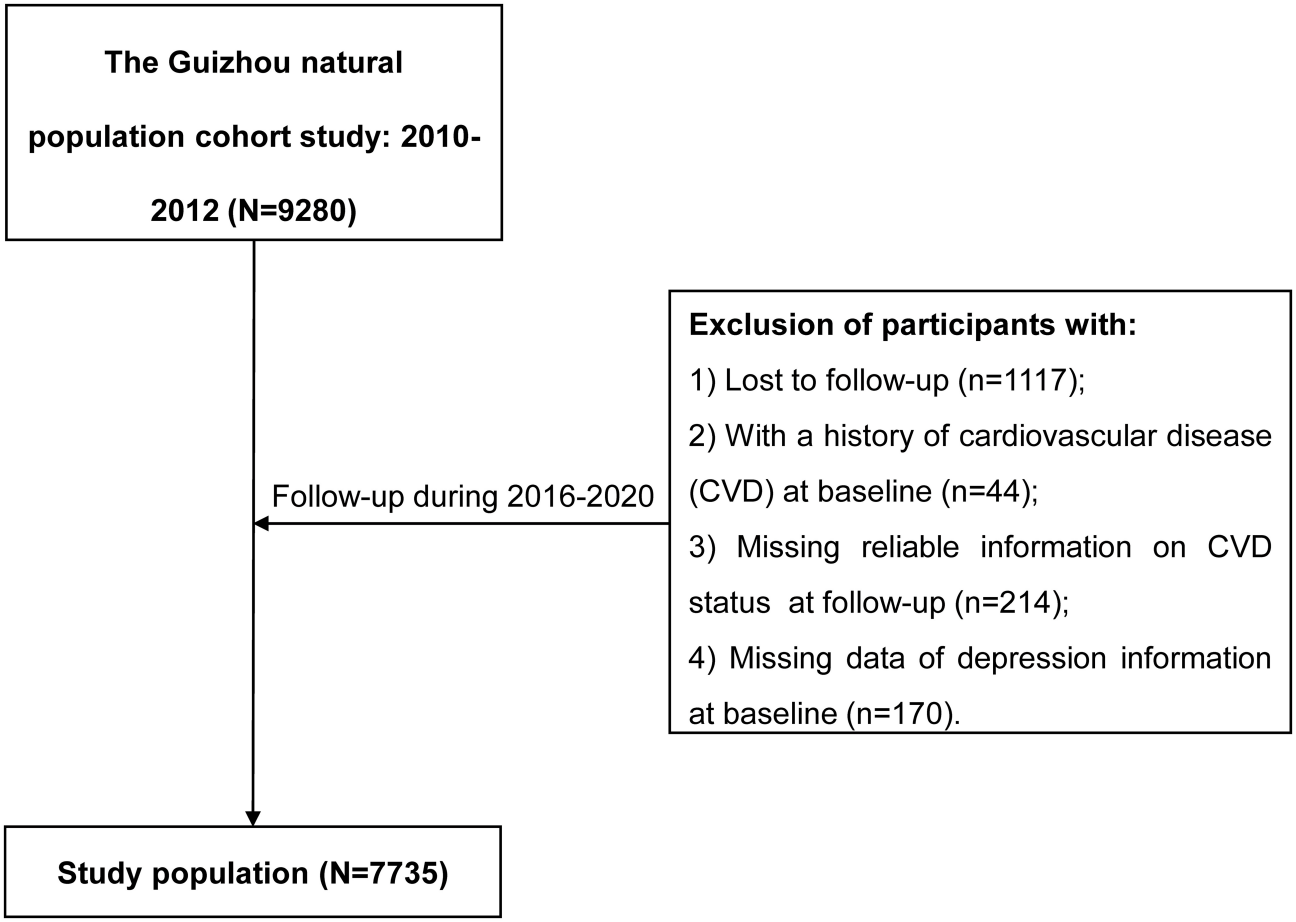
The flow chart.

Of 7,735 eligible subjects for the current analysis (Figure 1), 47.7% were men, with an average age of 44.37 ± 15.07 years old. A total of 500 (6.46%) participants was presented with depression (PHQ-9 score ≥ 5), with an average score of 7.22 ± 2.88, and near one-fifth (19.0%) had minimal depression with the PHQ-9 score between 1 to 4. Compared with participants without depression (PHQ-9 score = 0), depressive ones were older, women, ethnic minority, farmers, or having lower education levels (Table 1). They also had a lower proportion of current smokers and a higher prevalence of hypertension or dyslipidemia.

**Table 1:** General characteristics of the study population by the depression status at baseline in Southwest China.

	**Total** (***N*** **= 7,735)**	**Depression status**			***P-value***
		**No (0)**	**Minimal (1–4)**	**Mild or more advanced (≥5)**	
***N***	7,735	5,766	1,469	500	
**PHQ-9 score**	0.87 ± 2.05	0	2.11 ± 1.05	7.22 ± 2.88	<0.001
Age at baseline, years	44.37 ± 15.07	43.52 ± 14.95	46.80 ± 15.28	47.12 ± 14.78	<0.001
<30	1,517 (19.6)	1,213 (21.0)	236 (16.1)	68 (13.6)	<0.001
30.0–39.9	1,655 (21.4)	1,293 (22.4)	271 (18.4)	91 (18.2)	
40.0–49.9	1,942 (25.1)	1,433 (24.9)	369 (25.1)	140 (28.0)	
50.0–59.9	1,331 (17.2)	945 (16.4)	289 (19.7)	97 (19.4)	
60.0–69.9	824 (10.7)	569 (9.9)	190 (12.9)	65 (13.0)	
≥70.0	466 (6.0)	313 (5.4)	114 (7.8)	39 (7.8)	
Men, %	3,692 (47.7)	2,851 (49.4)	645 (43.9)	196 (39.2)	<0.001
Ethnic minority, %	3,197 (41.3)	2,471 (42.9)	541 (36.8)	185 (37.0)	<0.001
Education ≥9 years, %	3,328 (43.0)	2,618 (45.4)	544 (37.0)	166 (33.2)	<0.001
Married, %	6,251 (80.8)	4,631 (80.3)	1,224 (83.3)	396 (79.2)	0.021
Farmer, %	4,411 (57.0)	3,379 (58.6)	787 (53.6)	245 (49.0)	<0.001
Current smoker, %	1,972 (25.5)	1,502 (26.0)	364 (24.8)	106 (21.2)	0.045
Alcohol use, %^*^	1,525 (19.7)	1,146 (19.9)	299 (20.4)	80 (16.0)	0.089
Physical activity, %	67,41 (87.1)	5,023 (87.1)	1,311 (89.2)	407 (81.4)	<0.001
BMI, kg/m^2^	22.90 ± 3.36	22.90 ± 3.35	23.01 ± 3.46	22.55 ± 3.12	0.030
History of T2DM, % ^*^	657 (8.5)	486 (8.4)	130 (8.8)	41 (8.2)	0.202
History of hypertension, %	2,014 (26.0)	1,451 (25.2)	426 (29.0)	137 (27.4)	0.032
History of dyslipidemia, %	4,436 (57.3)	3,258 (56.5)	874 (59.5)	304 (60.8)	0.009

* *missing value*. *PHQ-9, Patient Health Questionnaire-9; BMI, body mass index; T2DM, type 2 diabetes mellitus*.

### Associations of Depression With Incident CVD, AMI, and Stroke

During the mean of 7.07 follow-up years, a total of 215 new CVD cases were identified with the crude incident density of 3.93 per 1,000 PYs, with 28 (0.51 per 1,000 PYs) new AMI cases and 197 (3.60 per 1,000 PYs) stroke cases (Table 2). The crude incident density was highest in the depression group (6.55 per 1,000 PYs), followed by minimal, and no depression groups. The age- and sex-adjusted Cox model showed that the PHQ-9 score was associated with an increased risk of incident CVD, AMI, and stroke. In the fully adjusted models, the adjusted HRs were 1.14 (95%CI: 1.03, 1.26) for CVD, 1.26 (95%CI: 1.01, 1.57) for AMI, and 1.12 (95%CI: 1.01, 1.25) for stroke with per SD increase of PHQ-9 score. Compared with no depression participants, those with minimal depression experienced a statistically increased risk of incident AMI, and those with depression had a higher risk of incident CVD and AMI.

**Table 2:** The incident risk of CVD, AMI, and Stroke associated with baseline depression status.

	**Cases, *n***	**Incident density/1000 PYs**	**Hazard Ratio (95% Confidence Interval)**	
			**Mode 1**	**Mode 2**
**CVD**				
**PHQ-9 score (per SD increase)**	215	3.93	1.14 (1.03, 1.26) ^*^	1.14 (1.03, 1.26) ^*^
No (0)	146	3.57	1.00	1.00
Minimal (1–4)	46	4.47	1.11 (0.79, 1.55)	1.10 (0.78, 1.53)
Mild or more advanced (≥5)	23	6.55	1.64 (1.05, 2.55) ^*^	1.69 (1.08, 2.64) ^*^
**AMI**				
**PHQ-9 score (per SD increase)**	28	0.51	1.26 (1.02, 1.55) ^*^	1.26 (1.01, 1.57) ^*^
No (0)	13	0.32	1.00	1.00
Minimal (1–4)	11	1.06	3.05 (1.36, 6.84) ^**^	3.11 (1.37, 7.07) ^**^
Mild or more advanced (≥5)	4	1.13	3.36 (1.09, 10.42) ^*^	3.36 (1.17, 10.56) ^*^
**Stroke**				
**PHQ-9 score (per SD increase)**	197	3.60	1.12 (1.00, 1.25) ^*^	1.12 (1.01, 1.25) ^*^
No (0)	137	3.35	1.00	1.00
Minimal (1–4)	40	3.89	1.02 (0.72, 1.46)	0.99 (0.70, 1.42)
Mild or more advanced (≥5)	20	5.69	1.51 (0.94, 2.42)	1.55 (0.96, 2.49)

*Model 1: adjusted for age (<30, 30–39, 40–49, 50–59, 60–69, ≥70), sex*.

*Model 2: model 1 plus ethnicity, education, marriage, occupation, smoking status, alcohol use, physical activity, history of T2DM, history of hypertension, history of dyslipidemia, and body mass index*.

** *P < 0.01*,

* *P < 0.05*.

*PY, person years; CVD, cardiovascular disease; PHQ-9, Patient Health Questionnaire-9; SD, standard deviation; AMI, acute myocardial infarction*.

In the sensitivity analysis (Table 3), the corresponding effect estimates of baseline depression status on the incident CVD and AMI did not change substantially after excluding participants who were diagnosed with CVD or AMI within 2 years after entering the cohort. However, the association between depression and incident stroke was attenuated, with the adjusted HR of 1.11 (95%CI: 0.99, 1.26) for per SD increase of PHQ-9 score. When the competing risk model was used to estimate the associations between depression with incident CVD, AMI, and stroke, the effects were similar to those in the main analysis.

**Table 3:** Sensitivity analysis.

	**Hazard Ratio (95% Confidence Interval)**		
	**CVD**	**AMI**	**Stroke**
**Excluding participants who were diagnosed within 2 years**			
PHQ-9 score (per SD increase)	1.13 (1.02, 1.26) ^*^	1.26 (1.01, 1.57) ^*^	1.11 (0.99, 1.26)
No (0)	1.00	1.00	1.00
Minimal (1–4)	1.21 (0.86, 1.72)	3.51 (1.53, 8.07) ^**^	1.12 (0.77, 1.63)
Mild or more advanced (≥5)	1.63 (1.00, 2.66) ^*^	3.62 (1.14, 11.47) ^*^	1.50 (0.88, 2.54)
**Competing risk model**			
PHQ-9 score (per SD increase)	1.13 (1.03, 1.24) ^**^	1.25 (1.04, 1.51) ^*^	1.12 (1.01, 1.24) ^*^
No (0)	1.00	1.00	1.00
Minimal (1–4)	1.09 (0.78, 1.52)	3.26 (1.42, 7.47) ^**^	1.00 (0.70, 1.42)
Mild or more advanced (≥5)	1.64 (1.06, 2.53) ^*^	3.26 (1.00, 10.67)	1.53 (0.96, 2.44)

*Adjusted for age (<30, 30–39, 40–49, 50–59, 60–69, ≥70), sex, ethnicity, education, marriage, occupation, smoking status, alcohol use, physical activity, history of T2DM, history of hypertension, history of dyslipidemia, and body mass index*.

** *:P < 0.01*,

* *P < 0.05*.

*CVD, cardiovascular disease; AMI, acute myocardial infarction; PHQ-9, Patient Health Questionnaire-9; SD, standard deviation*.

### Subgroup Analysis and Effect Modification

**Figure 2:**
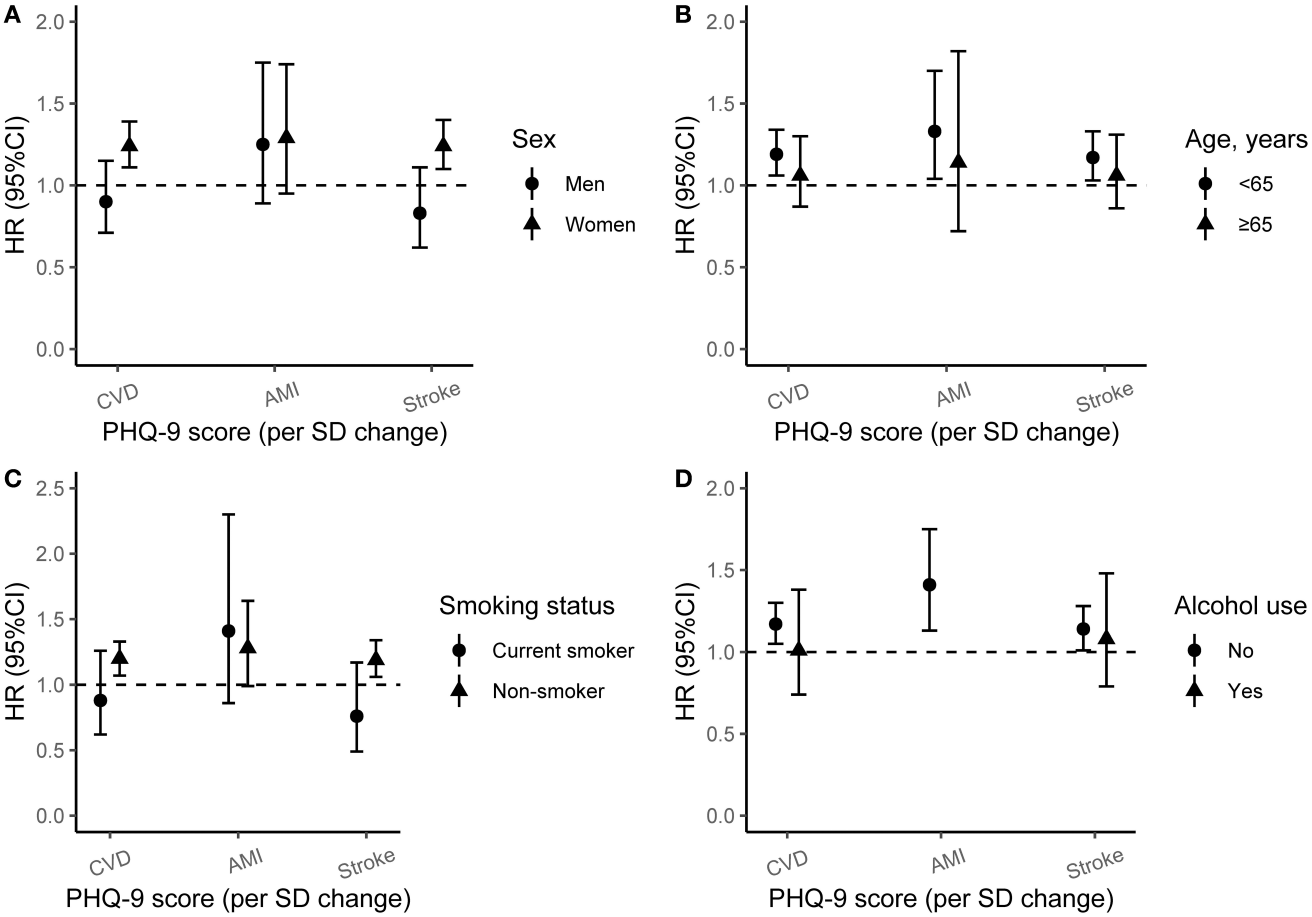
The incident risk of CVD, AMI, and Stroke associated with baseline PHQ-9 score by sex, age, smoking status, and alcohol use. Adjusted for age (<30, 30–39, 40–49, 50–59, 60–69, ≥70), sex, ethnicity, education, marriage, occupation, smoking status, alcohol use, physical activity, history of T2DM, history of hypertension, history of dyslipidemia, and body mass index. HR, hazard ratio; 95%CI, 95% confidence interval; PHQ-9, Patient Health Questionnaire-9; SD, standard deviation; CVD, cardiovascular disease; AMI, acute myocardial infarction. **(A)** Subgroup analysis by sex; **(B)** subgroup analysis by age group; **(C)** subgroup analysis by smoking status; **(D)** subgroup analysis by alcohol use.

We also explored the potential effect modification of baseline age, sex, smoking status, and alcohol use on the associations of depression with incident CVD, AMI, and stroke, and the results of the subgroup analyses were presented in Figure 2. The effects of PHQ-9 score on CVD and stroke were higher in women or participants aged <65 years than men or those aged ≥65 (*P* for the interaction of CVD and stroke <0.05). The associations between PHQ-9 score with stroke were stronger in non-smokers (*P* for the interaction <0.05). However, alcohol use modification was not significant.

## Discussion

This study examined the associations between depression and incident CVD, AMI, and stroke in a prospective cohort study of 7,735 adults in Southwest China with an average of 7 years follow-up. At baseline, 6.5% of the participants experienced a mild or more advanced depression. PHQ-9 score was associated with risk of incident CVD, AMI, and stroke, while depression was associated with 1.69-fold and 3.36-fold risks of CVD and AMI, respectively. Furthermore, we found that the associations were only significant in women, participants aged <65 years, non-smokers, and non-alcohol users.

Previous studies have suggested that depression is associated with an increased risk of CVD (2, 3, 7, 8, 16, 17). In the Jackson Heart Study among 3,309 participants followed-up for 10 years, O'Brien et al. found that major depressive symptoms, defined as a score of 21 or higher on the 20-item Center for Epidemiological Studies Depression Scale (CES-D), were associated with a 2-fold greater hazard of stroke, while a per-SD increase in CES-D score was associated with a 1.3-fold (16). In another cohort of the China Health and Retirement Longitudinal study among 12,417 middle-aged and older adults, Li et al. (8) reported that participants with elevated depressive symptoms had a 39% (95%CI: 22, 58%) higher risk of incident CVD, a 36% (95%CI: 18, 57%) higher risk of heart disease, and a 45% (95%CI: 6, 99%) higher risk of stroke during the 4 years of follow-up. A recent meta-analysis of 21 studies involving 47, 625 participants found that participants with depressive symptoms had a 1.36-fold higher risk of stroke, but not of MI (HR: 1.08, 95%CI: 0.91, 1.29) (18). As expected, the depressive symptoms were associated with a higher risk of CVD and AMI in this study, while the association was not statistically significant in stroke. Apart from differences in methods of depression symptoms assessment, different age distribution, the residual confounding effects, divergent medical, behavioral, or social responses to the depressive disorder may partly explain the different findings overstudies. Another potential explanation for such differences may be the limited number of new cases in this study. Thus, future work with a longer follow-up period is needed to confirm the association between depression and risk of CVD including AMI and stroke.

There are several potential mechanisms for the association of depression with excess risk of incident CVD. Biologically, depression is associated with hypothalamic-pituitary-adrenal axis hyperactivity (19), platelet activation (20), and immunological/inflammation effects (21), all of which might be linked to the increased CVD risk. Secondly, the depressive population often has unhealthy lifestyles, including smoking (22), alcohol abuse (23), low physical activity (24), and obesity (25), which could affect the occurrence of CVD. In addition, depression is associated with other comorbidities (26), like hypertension and diabetes, both are risk factors related to incident CVD. Nevertheless, after adjusting for baseline smoking status, alcohol use, physical activity, BMI, history of hypertension, and diabetes, the association between depression and incident CVD remained stable in this study, indicating that the effect of depression was independent of those risk factors mentioned above.

Sex modified associations between depression, and incident CVD and stroke in this study, which was inconsistent with several previous studies (4, 5, 8, 27). Those associations were more evident in women in this study. Hamano et al. evaluated sex differences in the association between depression and stroke and found that the effect of depression on stroke was higher in men compared with women (8). A meta-analysis of 17 prospective studies reported that the associations were similar between men and women (4). Even so, our findings provided new evidence that there may be a sex difference in the association of depression with incident CVD and stroke in the Chinese population. Although the exact mechanisms are still unclear, there are several potential biological and psychosocial explanations. First, the prevalence of depression is higher in women than men in this study. Compared with women, men may be less inclined to report a depressive disorder or seek help until the depression is severe (28, 29). Second, depression increases the plasma concentration of 5-hydroxytryptamine (5-HT), which is of particular relevance to women (20). 5-HT may affect platelet function and lead to platelet aggregation as well as coronary vasoconstriction (30). In addition, lifestyle differences may contribute to the stronger association in women. Previous studies have reported that there are several different risk factors associated with CVD in men and women, although the underlying biological mechanisms are still unclear (31, 32). The risk-elevating effect of depression on stroke tended to be preserved in a subgroup of non-smokers, which might be due to most smokers being men.

Previous cohort studies in the Chinese population were among middle-aged and older adults (7, 8), while this study included participants aged 18 years or above. In the stratified analysis by age, we found that the effects of depression on incident CVD and stroke were higher in participants aged <65 years, which was consistent with the previous study (6). The Framingham Study found that depression increased the risk of stroke in those aged <65 years but not in those aged ≥65 years. More prospective studies with a large sample size are calling to confirm whether age modifies the association between depression and incident CVD in the future.

The strengths of this study included the well-characterized prospective design and the longer follow-up period with a relatively low loss to follow-up rate. To our knowledge, this is the first report on the association between depression and incident CVD in different demographic groups in Southwest China. This study also had notable limitations. Firstly, we only measured baseline depression status using PHQ-9 and did not measure during the follow-up. Also, we did not have clinical diagnoses information of depression, which might lead to a misclassification of the depression status. Secondly, those with depressive disorders may be less likely to participate owing to their loss of interest in most things. Thirdly, the outcome was self-reported and the timing of onset may be inaccurate, and the association might be underestimated. In addition, even though current analyses adjusted for major potential confounding factors, residual confounding resulting from dietary factors was still possible.

In conclusion, depression significantly increased the risk of incident CVD including AMI and stroke, especially in women, participants aged <65 years, and non-smokers. Further prospective studies with clinically diagnosed depression and repeated measures of depression in Chinses population are required to examine the potential underlying mechanisms. Our findings highlight the need for focused attention on increasing awareness and improving the healthcare of depression, and also suggest that primary care physicians should pay more attention to CVD prevention if females, the elder, or non-smokers become depressed.

## Data Availability Statement

The raw data supporting the conclusions of this article will be made available by the authors, upon a reasonable request.

## Ethics Statement

The studies involving human participants were reviewed and approved by Institutional Review Board of Guizhou Center for Disease Control and Prevention. The patients/participants provided their written informed consent to participate in this study.

## Author Contributions

LY and YC: conceptualization, methodology, formal analysis, validation, writing—original draft, and visualization. NW and KX: conceptualization, methodology, data curation, writing—review, and editing. CW: methodology, data curation, writing—review, and editing. TL: conceptualization, methodology, supervision, funding acquisition, writing—review, and editing. CF: conceptualization, methodology, supervision, resources, writing—review, and editing. All authors contributed to the article and approved the submitted version.

## Funding

This work was supported by Guizhou Province Science and Technology Support Program [Grant number: Qiankehe (2018)2819].

## Conflict of Interest

The authors declare that the research was conducted in the absence of any commercial or financial relationships that could be construed as a potential conflict of interest.

## Publisher's Note

All claims expressed in this article are solely those of the authors and do not necessarily represent those of their affiliated organizations, or those of the publisher, the editors and the reviewers. Any product that may be evaluated in this article, or claim that may be made by its manufacturer, is not guaranteed or endorsed by the publisher.
